# Association of Sedentary Lifestyle with All-Cause and Cause-Specific Mortality in Adults with Reduced Kidney Function

**DOI:** 10.34067/KID.0000000000000313

**Published:** 2023-11-16

**Authors:** Min-Hsiang Chuang, Hung-Wei Wang, Yun-Ting Huang, Chung-Han Ho, Ming-Yan Jiang

**Affiliations:** 1 Renal Division, Department of Internal Medicine, Chi Mei Medical Center, Tainan, Taiwan; 2 Renal Division, Department of Internal Medicine, Chi Mei Hospital Chiali, Tainan, Taiwan; 3 Department of Medical Research, Chi Mei Medical Center, Tainan, Taiwan; 4 Department of Information Management, Southern Taiwan University of Science and Technology, Tainan, Taiwan; 5 Department of Pharmacy, Chia Nan University of Pharmacy and Science, Tainan, Taiwan; 6 Department of Public Health, College of Medicine, National Cheng Kung University, Tainan, Taiwan

**Keywords:** CKD, chronic kidney failure, health status, mortality, mortality risk

## Abstract

**Key Points:**

- Nearly half of individuals with reduced kidney function had sedentary lifestyle, defined as more than 6 hours of sitting a day.
- Non-Hispanic White individuals and individuals with younger age, obesity, and with diabetes were more likely to have sedentary lifestyles.
- Spending more than 6 hours a day sedentary increases the future risk of death from all causes and cardiovascular diseases in individuals with reduced kidney function.

**Background:**

Individuals with CKD tend to have sedentary behavior and decreased physical activity; both are independent predictors of mortality in general population. While physical inactivity correlates to adverse health outcomes in patients with reduced kidney function, it is unclear whether this relationship remains significant for sedentary behavior. Our study purpose was to evaluate the association of sedentary lifestyle with mortality risk in individuals with renal insufficiency.

**Methods:**

The study population were adult participants of 2007–2016 National Health and Nutrition Examination Survey with eGFR <60 ml/min per 1.73 m^2^ or self-reporting receiving dialysis (*N*=1419). Sedentary lifestyle was defined as sedentary time >6 hours per day. Outcome of interest was all-cause and cardiovascular disease (CVD)–related or cancer-related mortality.

**Results:**

We observed that non-Hispanic White individuals and individuals with younger age and higher educational level were more likely to have sedentary lifestyle. During a median follow-up of 99 (interquartile range, 70–128) months, a total of 458 participants died (3.98 deaths per 1000 person-months); 120 died from CVD and 92 from cancer, respectively. The crude analysis showed that individuals with sedentary lifestyle have higher risk of all-cause and CVD-related but not cancer-related mortality compared with the nonsedentary population. After adjusting for potential confounders, we showed that all-cause mortality and CVD-related mortality were 1.64-fold (95% confidence interval, 1.26 to 2.12) and 1.66-fold (95% confidence interval, 1.03 to 2.67) higher, respectively, in the sedentary population compared with the nonsedentary population. Similar results were observed in the sensitive analyses, in which we excluded individuals with dialysis, eGFR <15 ml/min per 1.73 m^2^, or mobility disability.

**Conclusions:**

Our findings suggest that sedentary lifestyle correlated to greater risk of all-cause and CVD-related mortality among individuals with reduced kidney function. Interventions targeting the individuals with risky behaviors may have practical importance for public health.

## Visual Abstract

**Figure:**
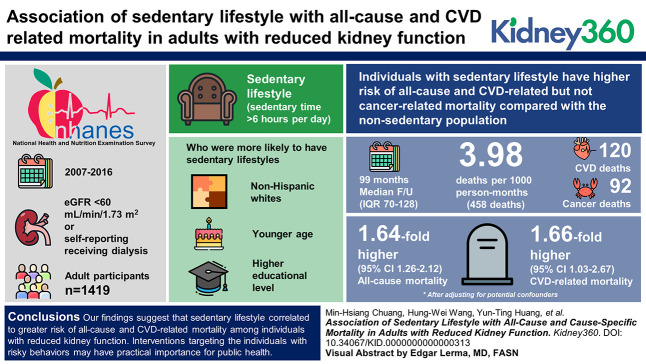

## Introduction

Sedentary behavior involves activities with low energy expenditure (*i.e.*, close to basal metabolic rate), such as watching television, using a computer, or sitting in an automobile.^1^ In the general population, sedentary behavior has been shown to be associated with increased risks of adverse health outcomes including cardiovascular disease (CVD), cancer, and all-cause mortality.^2–4^ Although high levels of moderate-intensity physical activity attenuate the increased risk of death associated with high sitting time, the detrimental association of sedentary behavior is not totally eliminated.^5^

CKD is associated with decreased physical activity^6^ and increased sedentary time during waking hours.^7^ Incidence and risk of death from CVDs and cancer are also increased in this population.^8,9^ On the other hand, sedentary behavior and physical inactivity are both independent risk factors of impaired renal function and incident ESKD.^10,11^ Current studies among patients with CKD mainly focused on physical activity, which was shown to be protective against adverse clinical outcomes and mortality risk.^6,12–15^ Research on the association of sedentary behavior with risk of death from all causes, CVDs, and cancer in this population is relatively lacking. Among incident dialysis patients, a previous study used the frequency of exercise to define sedentary behavior and showed that sedentary behavior, defined as self-reporting never or almost never exercised during leisure time, was associated with an increased risk of 1-year mortality.^16^ However, exercise frequency indicated physical activity, not sedentary behavior. Although sedentary behavior and physical inactivity are commonly considered to be composite variables in clinical research, they represent two different domains because people can engage in enough physical activity to meet public health guidelines but still spend most of their waking hours resting.^17^ Accordingly, our study aimed to investigate the association of sedentary behavior with all-cause and cause-specific mortality risk in adults with impaired renal function.

## Methods

### Data Source

Our data source is National Health and Nutrition Examination Survey (NHANES) of the United States, which is a series of health-related programs conducted by the National Center for Health Statistics (NCHS). NHANES constitutes a series of cross-sectional, multistage probability sampling for civilian noninstitutionalized population across the United States, collecting data from survey participants using questionnaires on health-related topics and a physical examination and laboratory tests. The data were released in 2-year cycles and were freely available on the website of NCHS (available from: https://wwwn.cdc.gov/nchs/nhanes/Default.aspx). All NHANES protocols were approved by the research ethics review board of the NCHS, and all the participants provided written informed consent. This study was adhered to the Declaration of Helsinki.

### Study Population

**Figure 1:**
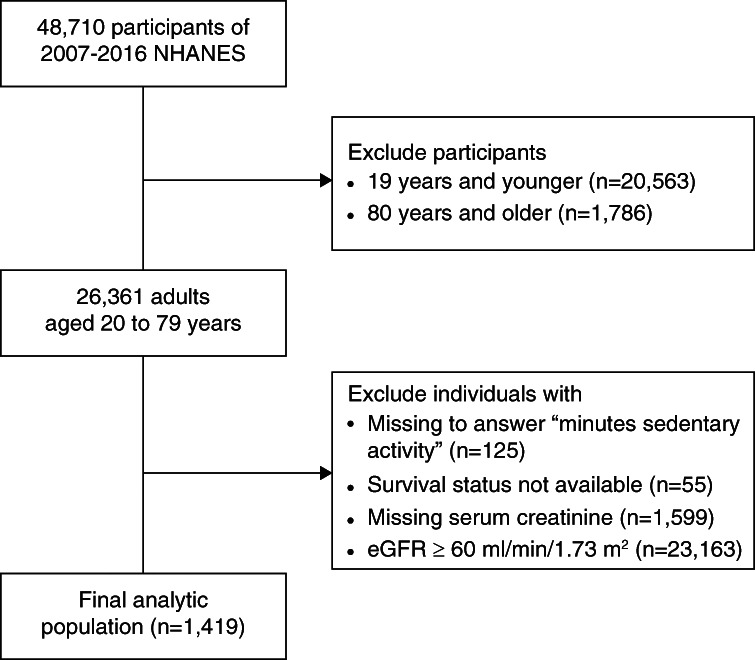
**Population selection flow chart.** NHANES, National Health and Nutrition Examination Survey.

In this retrospective cohort study, we merged the data from five discrete 2-year cycles (2007–2008 through 2015–2016) of the continuous NHANES and included the participants who underwent interviews and physical examinations (*n*=48,710). We excluded individuals younger than 19 years (*n*=20,563) or older than 80 years (*n*=1786) at the time of examination, those who missed to answer the question about minutes sedentary activity (*n*=125), whose survival status is not available (*n*=55), and those without serum creatinine data (*n*=1599) or with eGFR ≥60 ml/min per 1.73 m^2^ (*n*=23,163). Finally, we included 1419 individuals aged 20–79 years with reduced kidney function, which was defined as eGFR <60 ml/min per 1.73 m^2^ or self-reporting receiving dialysis in the past 12 months (Figure 1).

### Exposure

Minutes sedentary activity was ascertained from Physical Activity questionnaire in the NHANES, which is based on the Global Physical Activity Questionnaire. Participants were asked as following text: “The following question is about sitting at school, at home, getting to and from places, or with friends including time spent sitting at a desk, traveling in a car or bus, reading, playing cards, watching television, or using a computer. Do not include time spent sleeping. How much time do you usually spend sitting on a typical day?” Although there are no guidelines specifying how many hours of sitting a day may be harmful, previous studies have reported that spending more than 6 hours a day sedentary increases future risk of death from all causes and CVDs.^18–20^ Therefore, we defined those who self-report sitting for >360 minutes (6 hours) per day as sedentary and those who self-report ≤360 minutes (6 hours) as nonsedentary.

### Outcome

The outcomes of interest were death from all causes and from CVD or cancer. We linked the NHANES data to death records from the National Death Index to ascertain survival status through probabilistic matching and death certificate review. International Classification of Diseases, Tenth Revision, was used to define the cause of death. Deaths due to numerous causes were identified according to the leading causes of death included in the publicly available NHANES-linked mortality file. Death from CVD was defined by leading causes of death of I00–I09, I11, I13, and I20–I51 and from cancer as C00–C97. Participants were followed up from the NHANES baseline examination date to the participant's death date or last date of follow-up (December 31, 2019), whichever came first.

### Covariates

We categorized race/ethnicity by participants' self-report as non-Hispanic White participants, non-Hispanic Black participants, Hispanics, and other race including multiracial. Family income to poverty ratio was calculated by dividing total family income by the poverty threshold specific to family size and the appropriate year and state. Marital status was dichotomized into nonsingle (married or living with partner) and single (widowed, divorced, separated, or never married). Educational level was dichotomized into ≤high school graduate and some college or above. Diabetes and hypertension were defined by self-reporting diagnosis with the disease or taking medications. Body mass index (BMI) was calculated as body weight in kilograms divided by the square of height in meters. We classified BMI into three categories: <25, ≥25 to <30, and ≥30 kg/m^2^. The eGFR was calculated by CKD Epidemiology Collaboration 2021 equation.^21^ In addition, we selected items from the physical functioning questionnaire to define disabilities in mobility or activity of daily living (ADL).^22^ Mobility disability was assessed from two questions about difficulty walking for a quarter mile and walking up ten steps without resting. ADL disability was assessed from four questions asked about difficulty walking from one room to another on the same level, difficulty getting in and out of bed, difficulty eating, and difficulty dressing. We defined reporting some difficulty or above (some difficulty, much difficulty, or unable to do) in on one or more relevant tasks for that disability as presence of disability.^22^

### Statistical Analysis

The characteristics of the sample population were described using survey-weighted means and SEM or counts and survey-weighted proportions. Tests were two-tailed with a significance level of 0.05. Sample weights in NHANES have been constructed to adjust for nonresponse, oversampling, and noncoverage. We incorporated sampling weights, clustering, and stratification in regression analyses to account for the complex sampling design. We performed weighted Kaplan–Meier method with log-rank test to plot the survival curves. Weighted Cox regression analysis was performed to explore the association between sedentary lifestyle and mortality risk, with adjustment for age, sex, self-identified race/ethnicity, BMI (<25, 25–29.9, ≥30), diabetes, hypertension, smoking status, educational level, ratio of family income to poverty, and level of kidney function (eGFR 45–59.9, 30–44.9, 15–29.9, <15 ml/min per 1.73 m^2^, and dialysis). Data were presented as hazard ratio (HR) and 95% confidence interval (CI). The proportional hazards assumption was tested by log(−log[Survival]) plot and showed no violation of the assumption. In addition, we performed sensitivity analyses removing those with dialysis or eGFR <15 ml/min per 1.73 m^2^ and those with ADL or mobility disability. Furthermore, we used receiver operating characteristic (ROC) curve to identify the sedentary time that exhibited the maximum discriminatory power for predicting 5-year mortality. The optimal cutoff value was determined using Youden's index, which maximizes the sum of sensitivity and specificity, identifying the point on ROC curve that optimally balances the trade-off between true positives and true negatives. Statistical computation was performed using SAS 9.4.

## Results

The weighted age (mean±SEM) of the population was 65.8±0.4 years, and 41.5% of them were male, with race/ethnicity distribution of 67.5% White individuals, 20.7% Black individuals, and 7.3% Hispanics. We observed that nearly half of the population had sedentary lifestyle, similar in men and women (Table 1). When stratified by age groups, we found that more than half of people aged 65 years and younger had sedentary lifestyle, with a lower prevalence among people older than 65 years, 41.8%. In addition, while the prevalence of sedentary lifestyle in White individuals and Black individuals was close to 50%, it was lower in Hispanics and other races/ethnicities (Table 1). By logistic regression, we showed that the factors associated with sedentary lifestyle included younger age, non-Hispanic White individuals, single, higher BMI, diabetes, ADL disability, and mobility disability (Table 2).

**Table 1:** Survey-weighted characteristics of the sample population

Variables	Total	Nonsedentary	Sedentary
	*N*=1419	*N*=813 (54.0%)	*N*=606 (46.0%)
**Sex, *n* (%)**			
Male	665 (41.5)	369 (50.8)	296 (49.2)
Female	754 (58.5)	444 (56.3)	310 (43.7)
Age (years)	65.8±0.4	66.8±0.5	64.6±0.6
**Age group, *n* (%)**			
65 yr and younger	526 (39.9)	276 (47.6)	250 (52.4)
65 yr and older	893 (60.1)	537 (58.2)	356 (41.8)
**Race/ethnicity, *n* (%)**			
White	571 (67.5)	306 (51.5)	265 (48.5)
Black	543 (20.7)	301 (53.7)	242 (46.3)
Hispanic	230 (7.3)	155 (67.1)	75 (32.9)
Others	75 (4.4)	51 (71.7)	24 (28.3)
**Marital status^a^, *n* (%)**			
Nonsingle	760 (58.3)	450 (56.9)	310 (43.1)
Single	658 (41.7)	362 (49.9)	296 (50.1)
Missing (*N*)	1	1	0
**Educational level, *n* (%)**			
≤High school	804 (47.8)	480 (57.4)	324 (42.6)
≥Some college	611 (52.2)	330 (50.9)	281 (49.1)
Missing (*N*)	4	3	1
PIR	2.75±0.07	2.72±0.07	2.78±0.11
**PIR group, *n* (%)**			
<1.3	470 (26.1)	259 (52.3)	211 (47.7)
1.3 to <3.5	500 (38.4)	292 (54.2)	208 (45.8)
≥3.5	319 (35.5)	176 (51.0)	143 (49.0)
Missing (*N*)	130	86	44
BMI, kg/m^2^	30.8±0.3	29.6±0.3	32.3±0.5
**BMI group, *n* (%)**			
<25	275 (22.0)	173 (63.7)	102 (36.3)
25–29.9	420 (30.3)	274 (57.7)	146 (42.3)
≥30	685 (47.7)	351 (48.1)	334 (51.9)
Missing (*N*)	39	15	24
**Smoking status, *n* (%)**			
Never	645 (48.1)	379 (55.2)	266 (44.8)
Former	547 (37.8)	309 (52.9)	238 (47.1)
Current	226 (14.1)	124 (52.6)	102 (47.4)
Missing (*N*)	1	1	0
**Diabetes, *n* (%)**			
Yes	565 (35.1)	295 (46.1)	270 (53.9)
No	854 (64.9)	518 (58.3)	336 (41.7)
**Hypertension, *n* (%)**			
Yes	1121 (75.0)	636 (52.7)	485 (47.3)
No	298 (25.0)	177 (57.9)	121 (42.1
eGFR, ml/min per 1.73 m^2^^b^	48.7±0.4	49.2±0.5	48.2±0.7
**Level of kidney function, *n* (%)**			
eGFR 45–59.9	870 (66.6)	526 (57.2)	344 (42.8)
eGFR 30–44.9	342 (21.3)	192 (49.0)	150 (51.0)
eGFR 15–29.9	95 (5.5)	52 (55.2)	43 (44.8)
eGFR <15	22 (0.9)	5 (26.0)	17 (74.0)
Dialysis	90 (5.7)	38 (39.2)	52 (60.8)
**ADL disability, *n* (%)**			
Yes	443 (30.3)	216 (44.1)	227 (55.9)
No	850 (69.7)	532 (59.5)	318 (40.5)
Missing (*N*)	126	65	61
**Mobility disability, *n* (%)**			
Yes	357 (39.1)	203 (54.5)	154 (45.5)
No	544 (60.9)	367 (63.5)	177 (36.5)
Missing (*N*)	518	243	275

Note: Continuous variables were presented as weighted mean±SEM. Categorical variables were presented as count (weighted percentages), with row counts summing to 100%. There were varying amounts of missing data in each variable. For the categorical variables, the numbers were as indicated. For the continuous variables, there were no missing data in age and eGFR. For the variable ratio of family income to poverty, the total number of participants was 1,289, with 727 in the nonsedentary group and 562 in the sedentary group. For the variable body mass index, the total number of participants was 1,380, with 798 in the nonsedentary group and 582 in the sedentary group. ADL, activity of daily living; BMI, body mass index; eGFR: eGFR (in ml/min per 1.73 m^2^); PIR, ratio of family income to poverty.

a Nonsingle: married or living with partner; single: widowed, divorced, separate, or never married.

b Participants with dialysis were excluded.

**Table 2:** Factors associated with sedentary lifestyle by survey-weighted logistic regression

Variables	Crude OR (95% CI)	Age-Adjusted and Sex-Adjusted OR (95% CI)
**Sex**		
Male	1	—
Female	0.80 (0.59 to 1.09)	—
Age (every 10-yr increment)	0.83 (0.74 to 0.93)^a^	—
**Age group**		
65 yr and younger	1.53 (1.13 to 2.09)^a^	—
65 yr and older	1	—
**Race**		
White	1	1
Black	0.91 (0.70 to 1.20)	0.82 (0.61 to 1.09)
Hispanic	0.52 (0.37 to 0.72)^b^	0.42 (0.29 to 0.62)^b^
Others	0.42 (0.23 to 0.76)^a^	0.39 (0.20 to 0.75)^a^
**Marital status^c^**		
Nonsingle	1	1
Single	1.32 (0.95 to 1.84)	1.42 (1.03 to 1.97)^d^
**Educational level**		
≤High school	1	1
≥Some college	1.30 (0.99 to 1.72)	1.24 (0.94 to 1.65)
**PIR group**		
<1.3	1	1
1.3 to <3.5	0.93 (0.63 to 1.37)	1.01 (0.68 to 1.49)
≥3.5	1.05 (0.73 to 1.52)	1.03 (0.71 to 1.49)
BMI, kg/m^2^	1.05 (1.03 to 1.07)^b^	1.05 (1.03 to 1.07)^b^
**BMI group**		
<25	1	1
25–29.9	1.29 (0.80 to 2.07)	1.24 (0.78 to 1.99)
≥30	1.90 (1.31 to 2.74)^b^	1.85 (1.28 to 2.68)^a^
**Smoking status**		
Never	1	1
Former	1.10 (0.81 to 1.50)	1.13 (0.82 to 1.56)
Current	1.11 (0.77 to 1.60)	1.08 (0.76 to 1.53)
Diabetes	1.63 (1.24 to 2.14)^b^	1.64 (1.24 to 2.16)^b^
Hypertension	1.24 (0.89 to 1.72)	1.33 (0.95 to 1.87)
eGFR (every ten-unit increment)^c^	0.91 (0.79 to 1.05)	0.88 (0.76 to 1.01)
**Level of kidney function**		
eGFR 45–59.9	1	1
eGFR 30–44.9	1.39 (0.99 to 1.95)	1.50 (1.07 to 2.09)^d^
eGFR 15–29.9	1.08 (0.62 to 1.91)	1.12 (0.64 to 1.97)
eGFR <15	3.79 (1.21 to 11.86)^d^	3.63 (1.12 to 11.81)^d^
dialysis	2.07 (1.05 to 4.07)^d^	1.75 (0.86 to 3.55)
ADL disability	1.86 (1.29 to 2.69)^a^	1.80 (1.23 to 2.63)^a^
Mobility disability	1.45 (1.00 to 2.11)^d^	1.48 (1.01 to 2.17)^d^

ADL, activity of daily living; BMI, body mass index; CI, confidence interval; eGFR, eGFR (in ml/min per 1.73 m^2^); OR, odds ratio; PIR, ratio of family income to poverty.

a *P* < 0.01.

b *P* < 0.001.

c Participants with dialysis were excluded.

d *P* < 0.05.

**Figure 2:**
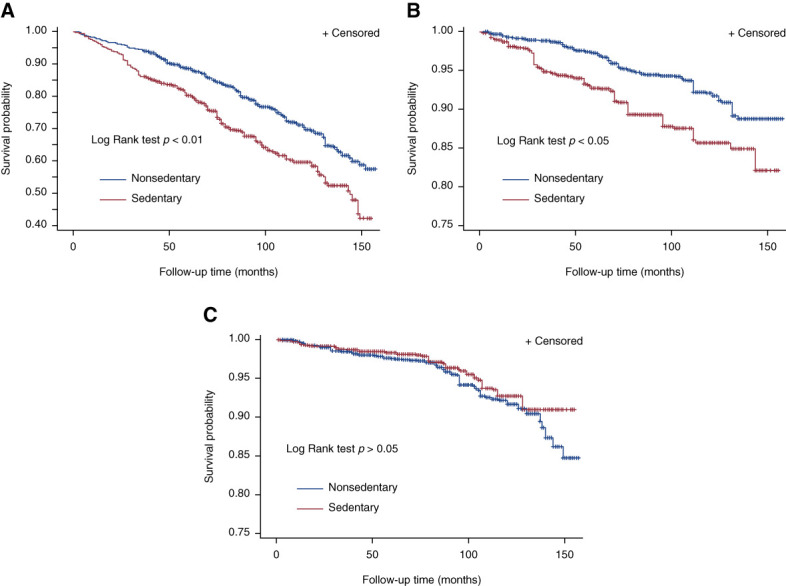
**Sedentary lifestyle is associated with increased risk of death from all causes and from CVD.** Survival curves for (A) all-cause mortality (weighted log-rank test *P* < 0.01), (B) CVD-related mortality (weight log-rank test *P* < 0.05), and (C) cancer-related mortality (weighted log-rank test *P* > 0.05). CVD, cardiovascular disease.

**Figure 3:**
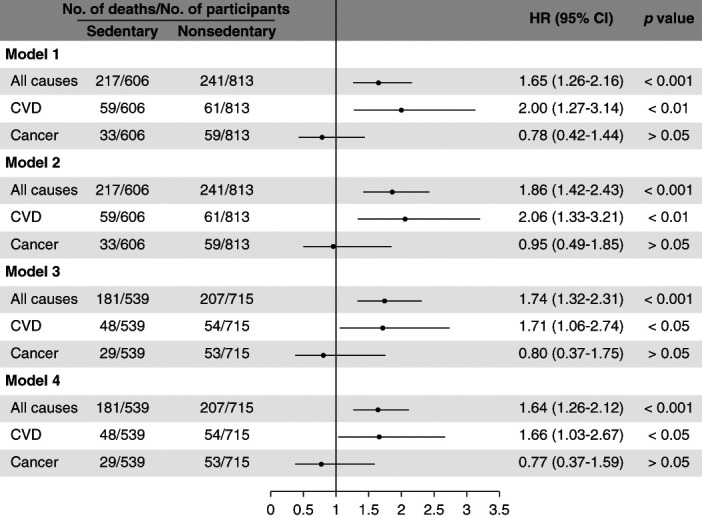
**Risk of death from all causes, CVD, and cancer in the sedentary population compared with the nonsedentary population.** Model 1: crude model. Model 2: adjusted for age and sex. Model 3: adjusted for age, sex, race/ethnicity (category), BMI (category), diabetes, hypertension, smoking status (category), educational level, family income to poverty ratio (continuous). Model 4: adjusted for age, sex, race/ethnicity (category), BMI (category), diabetes, hypertension, smoking status (category), educational level, family income to poverty ratio (continuous), and level of kidney function (category). Note: The sum of participants for regression models 3 and 4 does not equal the total because of missing data. BMI, body mass index; CI, confidence interval; HR, hazard ratio.

During a median follow-up of 99 (interquartile range, 70–128) months, 458 participants died (3.98 deaths per 1000 person-months), of whom 120 died from CVD and 92 died from cancer. The crude analyses showed that individuals with sedentary lifestyle were at higher risk for all-cause and CVD-related mortality, but not cancer-related mortality (Figure 2 and model 1 in Figure 3). After adjusting for age and sex, we observed a 1.86-fold (95% CI, 1.42 to 2.43) and 2.06-fold (95% CI, 1.33 to 3.21) higher risk of death from all causes and from CVD, respectively, in the sedentary population compared with the nonsedentary population (Figure 3). After further adjusting for other potential confounders, our results showed that sedentary lifestyle correlated to greater risk of all-cause and CVD-related mortality (Figure 3). In addition, we also observed a significant association between sedentary lifestyle and 5-year mortality risk (Supplemental Table 1).

In the sensitivity analyses, we observed that sedentary lifestyle was associated with an increased risk of all-cause mortality, but not CVD-related or cancer-related mortality when we excluded individuals with dialysis, eGFR <15 ml/min per 1.73 m^2^, or mobility disability from the study population (Supplemental Figure 1, A–C). When we excluded individuals with mobility disability or ADL disability, the association between sedentary lifestyle and mortality risk attenuated (Supplemental Figure 1D).

**Figure 4:**
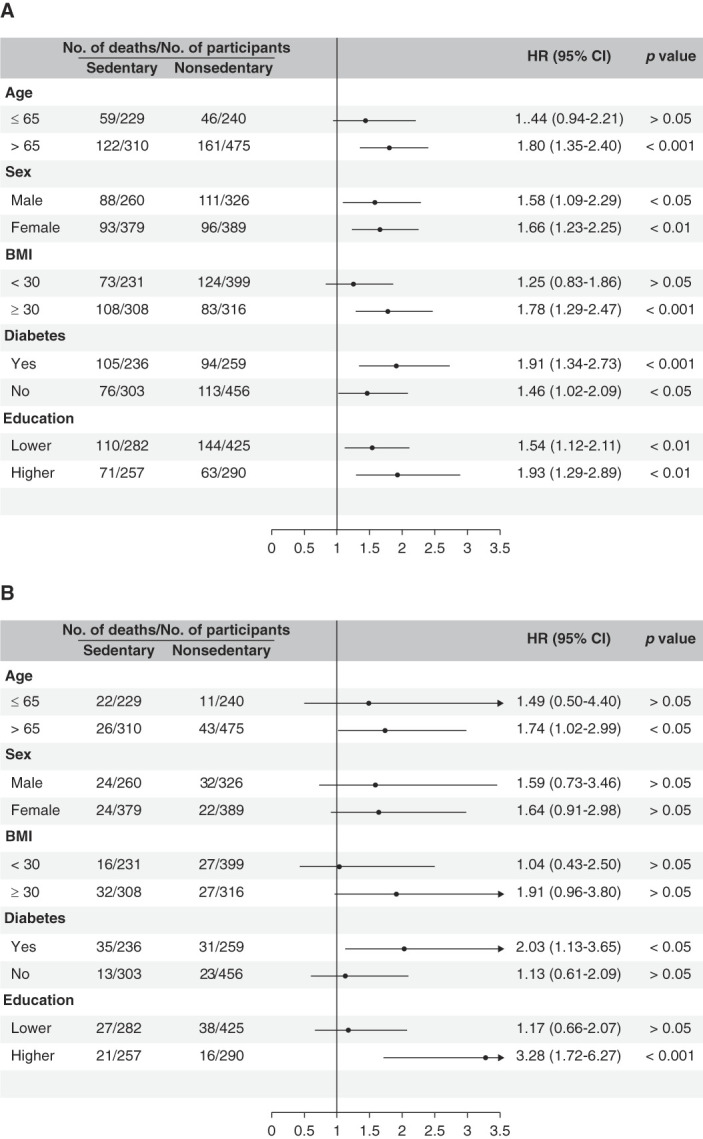
**Stratified analysis.** Risk of death from (A) all causes and from (B) CVD in the sedentary population compared with the nonsedentary population stratified by age, sex, BMI, diabetes, and educational level. The regression model was adjusted for age, sex, race/ethnicity (category), BMI (category), diabetes, hypertension, smoking status (category), educational level, family income to poverty ratio (continuous), and level of kidney function (category). Note: lower educational level: ≤high school; higher educational level: ≥some college.

In the stratified analyses (Figure 4, A and B), we observed a significant association between sedentary lifestyle and risk of all-cause mortality (HR, 1.80; 95% CI, 1.35 to 2.40) or CVD-related mortality (HR, 1.74; 1.02 to 2.99) in people older than 65 years, but not in those aged 65 years and younger. When stratified by sex, our results suggested that sedentary lifestyle was associated with increased risk of all-cause mortality in both men (HR, 1.58; 1.09 to 2.29) and women (HR, 1.66; 1.23 to 2.25); the risk of CVD-related death also increased in both sexes, but did not reach statistical significance. In addition, the association between sedentary lifestyle and all-cause mortality was significant among those with BMI ≥30, with or without diabetes, and with lower (≤high school) or higher (≥some college) educational attainment. Furthermore, we observed a significant association between sedentary lifestyle and CVD-related mortality among those with diabetes and with higher educational attainment (Figure 4, A and B).

**Figure 5:**
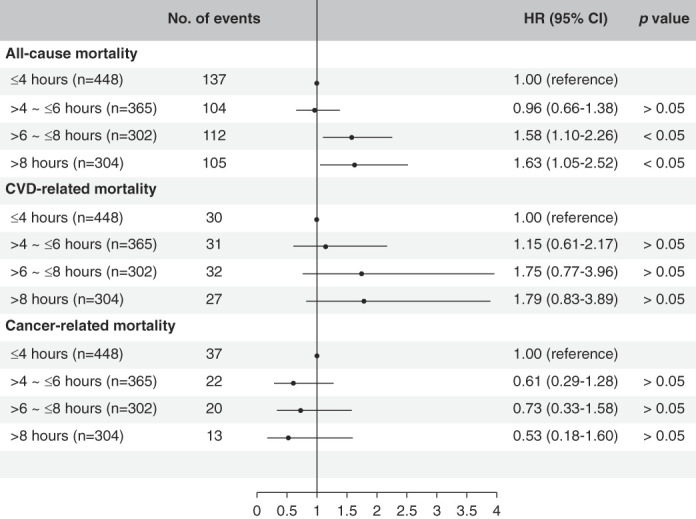
**Risk of death from all causes, CVD, and cancer among participants with 4–6, 6–8 hours, and more than 8 hours sedentary a day compared with those with <4 hours sedentary a day.** The regression model was adjusted for age, sex, race/ethnicity (category), BMI (category), diabetes, hypertension, smoking status (category), educational level, family income to poverty ratio (continuous), and level of kidney function (category).

When dividing the participants by sedentary time into four groups (Figure 5), we showed that individuals with sedentary time 6–8 hours (HR, 1.58; 95% CI, 1.10 to 2.26) and more than 8 hours per day (HR, 1.63; 95% CI, 1.05 to 2.52) had higher risk of all-cause mortality when compared with those with sedentary time <4 hours per day, but the association in CVD-related or cancer-related mortality was not significant.

By ROC curve approach with Youden's index, we found that a cutoff of 8 hours of sitting time best discriminated 5-year mortality in this population. When using this cutoff (8 hours) to examine the association between sedentary lifestyle and 5-year mortality risk (Supplemental Table 2), we observed similar results with the original analysis (6-hour cutoff). However, the area under the curve indicated a better accuracy for the 6-hour than the 8-hour cutoff values (Supplemental Table 3).

## Discussion

Among noninstitutionalized US adults with reduced kidney function, nearly half of them had sedentary lifestyle, defined as sedentary activity more than 6 hours per day. In addition, we observed that non-Hispanic White individuals and individuals with younger age, higher BMI, and with diabetes were more likely to have sedentary lifestyle. Furthermore, our results showed that sedentary lifestyle correlated to 1.64-fold and 1.66-fold greater risk of death from all causes and from CVDs, respectively, when compared with those without sedentary lifestyle.

The association between sedentary time and mortality risk in patients with impaired renal function had been evaluated in several studies. Among a cohort of 71 Japanese hemodialysis patients, a study showed that longer sedentary time correlated to higher all-cause mortality risk.^23^ In addition, among nondialysis patients with CKD, extended sedentary time was associated with risk of all-cause and CVD-related mortality.^24,25^ These studies reported a linear association between sedentary time and mortality risk, which was questionable. A systematic review demonstrated a nonlinear association between total sitting time and all-cause mortality in general population, with a threshold identified at 3–4 hours per day of TV viewing, above which the risk was significantly increased.^19^ Our study added evidence to support the association between nonlinear relationship, showing that sedentary time of more than 6 hours per day was associated with increased risk of death.

Several studies had demonstrated the correlations between sedentary time and CVD mortality among the general population^2,26^; the results were generally consistent and in line with our findings. On the other hand, the relationship between sedentary behavior and cancer-related mortality was less well-defined. While some studies found a significant association,^18,27,28^ others produced null association.^29,30^ The association was reported linear for TV viewing time but nonsignificant for total sedentary time in one systematic review with dose-response meta-analysis,^19^ whereas a J-shaped nonlinear correlation for total sedentary time was shown in another study.^31^ There is still substantial uncertainty surrounding the association between sedentary behavior and risk of death from cancer, and further research is therefore much needed.

Sedentary behavior increased the burden of various chronic diseases and physiologic impairments, which could ultimately lead to adverse health outcomes. Musculoskeletal disorders associated with sedentary lifestyles included osteoporosis,^32^ joint disorders,^33^ and decreased skeletal muscle strength,^34,35^ which was an independent risk factor for all-cause mortality.^36,37^ As for metabolic and circulatory derangements, sedentary time was found to increase the risk of several known CVD risk factors including diabetes mellitus, dyslipidemia, and hypertension.^2,38–40^ Proposed mechanisms underlying the pathologic process included microvascular dysfunction, alterations in lipoprotein metabolism, insulin insensitivity, and increased adiposity.^38,41,42^ Prolonged sitting for 3 hours was found to cause an impairment in vascular function, which could be prevented with 5 minutes of light activity at hourly intervals.^43^ Of note, our study demonstrated that all-cause and CVD-related mortality remained elevated after adjustment for diabetes, hypertension, and BMI, indicating that these disorders themselves were not fully explanatory of the increased risk of death, and other mediating factors might have existed and remained to be explored.

The World Health Organization 2020 guidelines on physical activity and sedentary behavior recommended that all adults should limit the amount of sedentary time and replace it with physical activity of any intensity, although the current evidence was insufficient to provide specific thresholds of sedentary behavior, to determine whether the effect varied between different kinds of sedentary activities, or to determine the influence of frequency and duration of breaks.^44^ While recommendations on physical activity and sedentary behavior for CKD population were not provided in the WHO guideline,^44^ our study supported the benefit of minimizing sedentary time in patients with CKD and suggested that reducing sedentary time to <6 hours per day might be a reasonable goal that conferred a survival benefit in this population. In addition, our study identified the subpopulation who were more likely to have sedentary lifestyle among patients with CKD, which may have important implications for public health policy. Of note, we found that non-Hispanic White individuals were more likely to have sedentary lifestyle, while the Hispanics were less likely; this observation is consistent with a previous study in the US general population.^45^ This difference between racial/ethnic groups may be due to the disparity in socioeconomic status, in which higher educational attainment and higher household income were found to be significantly associated with more daily sitting time.^45,46^ Nonetheless, there is currently a lack of research on the interaction of socioeconomic factors and sedentary behavior on clinical outcomes, which remains to be answered in future studies.

Our study had some limitations that should be considered. First, the sitting time data were self-reported instead of being recorded by devices. Although self-reported data effectively avoided misclassification and incorrect estimation of sedentary time commonly seen with accelerometry,^47^ it might still not reflect the precise behavioral pattern of the participants because of recording errors, recall bias, and social desirability bias. Second, the sedentary time was recorded as total sitting minute per day, so the effects of its component activities (*e.g.*, during commuting, watching television, at work, *etc.*) and whether they differ among each other remained to be addressed. Third, sedentary time was collected at baseline and might fail to capture behavioral changes during the follow-up period, especially in our study where the length of follow-up varied widely. However, our results remained similar when using 5-year mortality as the study outcome. Fourth, residual confounding that was hard to be accurately specified (*e.g.*, family history of cardiovascular diseases, environmental exposures, or dietary habits) might have biased our results. For example, our sensitivity analysis showed no significant association between sedentary behavior and mortality risk among those without ADL or mobility disability. While the substantial reduction in sample size may have affected statistical power, confounding factors such as healthy lifestyle (*e.g.*, physical activity and eating habits) not included in our study may have contributed to the null results. On the other hand, given the lack of a dose-dependent association between sedentary time and mortality risk, it is also possible that the 6-hour threshold simply differentiates between those with and without ADL or mobility disability. Finally, although this study used a large, ethnically diverse data from the NHANES of the United States, our findings may not be fully extrapolatable to other places with different health care systems and ethnic composition.

In summary, our study indicated that sedentary lifestyle was associated with increased risk of all-cause and CVD-related mortality among individuals with reduced kidney function. In addition, we observed that non-Hispanic White individuals and individuals with younger age and higher BMI were more likely to have sedentary lifestyle. Our findings have practical importance for public health because interventions targeting individuals with risky behaviors may help reduce adverse health outcomes.

## Supplementary Material

SUPPLEMENTARY MATERIAL

## Data Availability

Anonymized data created for the study are available in NHANES website. https://wwwn.cdc.gov/nchs/nhanes/Default.aspx.

## Supplemental Material

Supplemental Table 1. Five-year risk of death from all causes, cardiovascular disease (CVD), and cancer in the sedentary population compared with the nonsedentary population.

Supplemental Table 2. Five-year risk of death from all causes, cardiovascular disease (CVD), and cancer in people with sedentary lifestyle, defined as sitting for more than 8 hours a day, compared with those without sedentary lifestyle.

Supplemental Table 3. Performance of 6-hour (360 minutes a day) and 8-hour (480 minutes a day) cutoff value of sitting time to predictive 5-year mortality risk.

Supplemental Figure 1. Sensitivity analysis. Risk of death from all causes, cardiovascular disease (CVD), and cancer in the sedentary population compared with the nonsedentary population when excluding participants with (A) dialysis (*n*=90), (B) eGFR <15 ml/min per 1.73 m^2^ or dialysis (*n*=112), (C) mobility disability (*n*=357), and (D) mobility or ADL disability (*n*=677). Model 1: crude model. Model 2: adjusted for age and sex. Model 3: adjusted for age, sex, race/ethnicity (category), body mass index (category), diabetes, hypertension, smoking status (category), educational level, family income to poverty ratio (continuous). Model 4: adjusted for age, sex, race/ethnicity (category), body mass index (category), diabetes, hypertension, smoking status (category), educational level, family income to poverty ratio (continuous), and level of kidney function (as continuous [eGFR] in [A] and [B] and as categorical [eGFR 45–59.9, 30–44.9, 15–29.9, <15 ml/min per 1.73 m^2^, and dialysis] in [C] and [D]). Note: (*1*) The average sedentary time (survey mean±SEM) among individuals with mobility disability, ADL disability, and mobility or ADL disability were 396.9 (±12.9), 467.2 (±15.2), and 441.9 (±11.6) minutes, respectively. (*2*) The sum of participants for regression models 3 and 4 does not equal the total because of missing data.
