# Supplementary material for: Association of Sedentary Lifestyle with All-Cause and Cause-Specific Mortality in Adults with Reduced Kidney Function
Source: Kidney360. 2023 Nov 16;5(1):33–43. doi: 10.34067/KID.0000000000000313 (PMC10833594; doi:10.34067/KID.0000000000000313)

Association of sedentary lifestyle with all-cause and cause-specific mortality in adults with reduced kidney function

Min-Hsiang Chuang M.D. [1], Hung-Wei Wang M.D. [2], Yun-Ting Huang M.D. [2], Chung-Han Ho Ph.D. [3,4], Ming-Yan Jiang M.D., MSc [1,5,6]

[1] Renal division, Department of Internal Medicine, Chi Mei Medical Center, Tainan, Taiwan

[2] Renal division, Department of Internal Medicine, Chi Mei Hospital Chiali, Tainan, Taiwan

[3] Department of Medical Research, Chi Mei Medical Center, Tainan, Taiwan

[4] Department of Information Management, Southern Taiwan University of Science and Technology, Tainan, Taiwan

[5] Department of Pharmacy, Chia Nan University of Pharmacy & Science, Tainan, Taiwan

[6] Department of Public Health, College of Medicine, National Cheng Kung University, Tainan, Taiwan

Table of Contents for the Supplemental Material

Supplementary Table S1. Five-year risk of death from all causes, cardiovascular disease (CVD), and cancer in the sedentary population compared with the non-sedentary population

Supplementary Table S2. Five-year risk of death from all causes, cardiovascular disease (CVD), and cancer in people with sedentary lifestyle, defined as sitting for more than 8 hours a day, compared with those without sedentary lifestyle

Supplementary Table S3. Performance of 6-hour (360 minutes a day) and 8-hour (480 minutes a day) cutoff value of sitting time to predictive 5-year mortality risk

Supplementary Figure S1. Sensitivity analysis. Risk of death from all causes, cardiovascular disease (CVD), and cancer in the sedentary population compared with the non-sedentary population when excluding participants with (A) dialysis (n=90), (B) eGFR < 15 ml/min/1.73 m^2 or dialysis (n=112), (C) mobility disability (n=357), and (D) mobility or ADL disability (n=677).

Supplementary Table S1. Five-year risk of death from all causes, cardiovascular disease (CVD), and cancer in the sedentary population compared with the non-sedentary population

	No. of events	Model 1	Model 2	Model 3	Model 4
		HR (95% CI)	HR (95% CI)	HR (95% CI)	HR (95% CI)
All-cause mortality					
Non-sedentary (n=813)	113	1	1	1	1
Sedentary (n=606)	135	1.84 (1.26-2.69) **	1.95 (1.35-2.83) ***	1.74 (1.21-2.50) **	1.62 (1.20-2.17) **
CVD-related mortality					
Non-sedentary (n=813)	29	1	1	1	1
Sedentary (n=606)	45	2.79 (1.54-5.06) **	2.83 (1.57-5.08) ***	2.14 (1.17-3.93) *	2.05 (1.15-3.65) *
Cancer-related mortality					
Non-sedentary (n=813)	26	1	1	1	1
Sedentary (n=606)	16	0.69 (0.32-1.49)	0.71 (0.25-2.04)	0.71 (0.25-2.04)	0.71 (0.28-1.81)

Model 1: crude model

Model 2: adjusted for age and sex.

Model 3: adjusted for age, sex, race/ethnicity (category), body mass index (category), diabetes, hypertension, smoking status (category), educational level, family income to poverty ratio (continuous).

Model 4: adjusted for age, sex, race/ethnicity (category), body mass index (category), diabetes, hypertension, smoking status (category), educational level, family income to poverty ratio (continuous), and level of kidney function (category)

*: $p<0.05$; **: $p<0.01$; ***: $p<0.001$.

Supplementary Table S2. Five-year risk of death from all causes, cardiovascular disease (CVD), and cancer in people with sedentary lifestyle, defined as sitting for more than 8 hours a day, compared with those without sedentary lifestyle

	Model 1	Model 2	Model 3	Model 4
	HR (95% CI)	HR (95% CI)	HR (95% CI)	HR (95% CI)
Total population	1.64 (1.00-2.68)	1.75 (1.08-2.82) *	1.71 (1.09-2.69) *	1.70 (1.13-2.56) *
Stratified by sex				
Male	2.01 (1.11-3.67) *	2.17 (1.18-3.98) *	1.94 (0.91-4.13)	1.85 (1.00-3.42)
Female	1.36 (0.71-2.63)	1.45 (0.77-2.71)	1.53 (0.87-2.69)	1.58 (0.91-2.76)
Stratified by age				
≤ 65 years old	1.14 (0.47-2.76)	1.11 (0.46-2.70)	1.08 (0.60-1.95)	0.96 (0.54-1.73)
> 65 years old	2.15 (1.23-3.77) **	2.20 (1.27-3.81) **	2.04 (1.21-3.43) **	1.96 (1.21-3.18) **

Model 1: crude model

Model 2: adjusted for age and sex.

Model 3: adjusted for age, sex, race/ethnicity (category), body mass index (category), diabetes, hypertension, smoking status (category), educational level, family income to poverty ratio (continuous).

Model 4: adjusted for age, sex, race/ethnicity (category), body mass index (category), diabetes, hypertension, smoking status (category), educational level, family income to poverty ratio (continuous), and level of kidney function (category)

*: p<0.05; **: p<0.01; ***: p<0.001.

Supplementary Table S3. Performance of 6-hour (360 minutes a day) and 8-hour (480 minutes a day) cutoff value of sitting time to predictive 5-year mortality risk

	Cutoff value of sitting time	Sensitivity	Specificity	AUC	*p* value #
Original	360 minutes a day	0.61	0.45	0.5345	0.0497
By Youden index score	480 minutes a day	0.45	0.64	0.5111	

AUC: area under the curve

#: *p* value between the two AUC

Supplemental Figure S1A

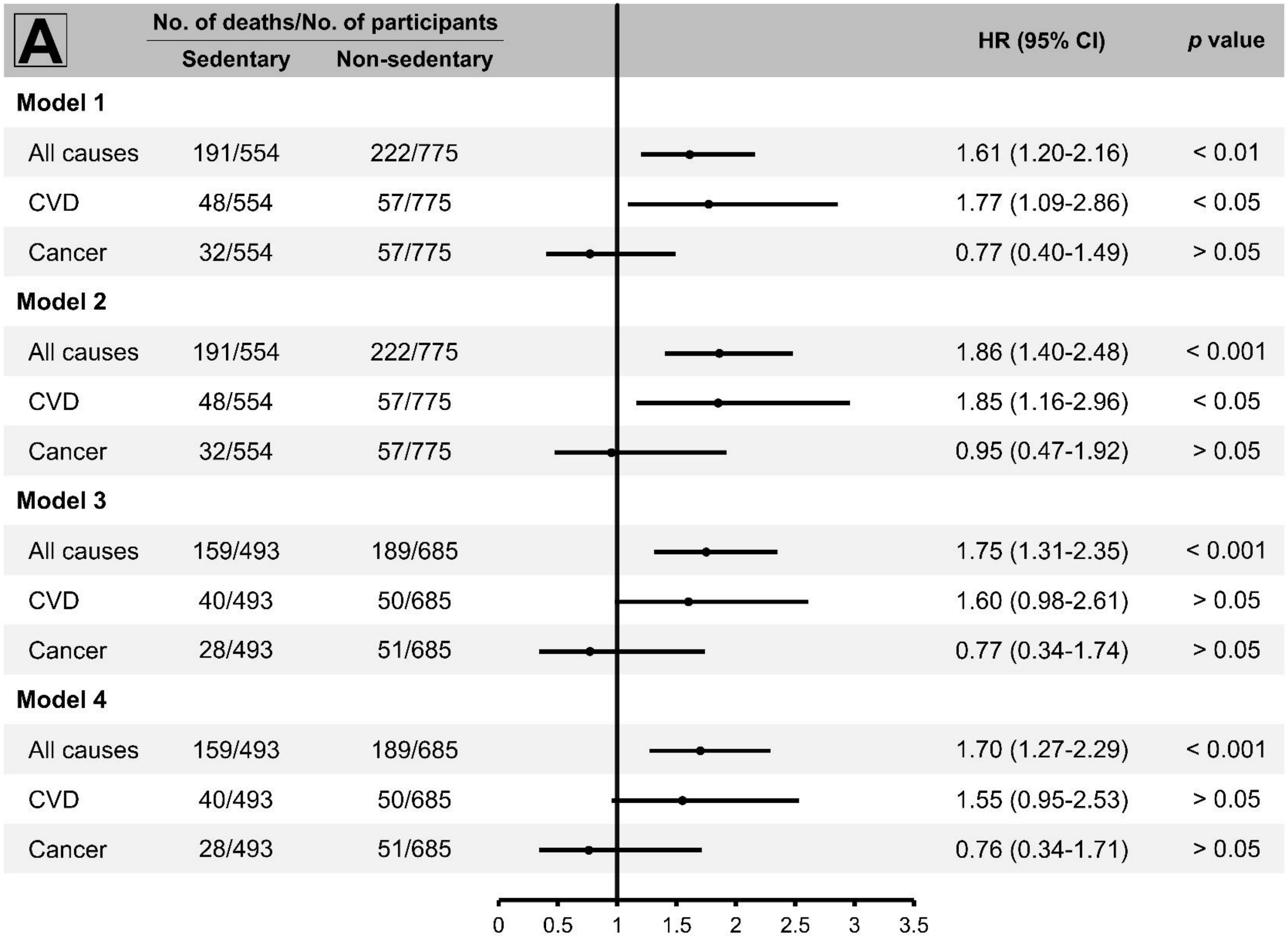

	No. of deaths/No. of participants		HR (95% CI)	*p* value
	Sedentary	Non-sedentary		
**Model 1**				
All causes	191/554	222/775	1.61 (1.20-2.16)	< 0.01
CVD	48/554	57/775	1.77 (1.09-2.86)	< 0.05
Cancer	32/554	57/775	0.77 (0.40-1.49)	> 0.05
**Model 2**				
All causes	191/554	222/775	1.86 (1.40-2.48)	< 0.001
CVD	48/554	57/775	1.85 (1.16-2.96)	< 0.05
Cancer	32/554	57/775	0.95 (0.47-1.92)	> 0.05
**Model 3**				
All causes	159/493	189/685	1.75 (1.31-2.35)	< 0.001
CVD	40/493	50/685	1.60 (0.98-2.61)	> 0.05
Cancer	28/493	51/685	0.77 (0.34-1.74)	> 0.05
**Model 4**				
All causes	159/493	189/685	1.70 (1.27-2.29)	< 0.001
CVD	40/493	50/685	1.55 (0.95-2.53)	> 0.05
Cancer	28/493	51/685	0.76 (0.34-1.71)	> 0.05

Supplemental Figure S1B

B	No. of deaths/No. of participants		HR (95% CI)	*p* value
	Sedentary	Non-sedentary		
**Model 1**				
All causes	184/537	219/770	1.62 (1.20-2.17)	< 0.01
CVD	45/537	57/770	1.74 (1.08-2.81)	< 0.05
Cancer	32/537	56/770	0.79 (0.41-1.53)	> 0.05
**Model 2**				
All causes	184/537	219/770	1.87 (1.41-2.49)	< 0.001
CVD	45/537	57/770	1.83 (1.15-2.90)	< 0.05
Cancer	32/537	56/770	0.97 (0.48-1.97)	> 0.05
**Model 3**				
All causes	154/479	186/680	1.78 (1.32-2.39)	< 0.001
CVD	37/479	50/680	1.57 (0.97-2.55)	> 0.05
Cancer	28/479	50/680	0.79 (0.35-1.81)	> 0.05
**Model 4**				
All causes	154/479	186/680	1.76 (1.31-2.36)	< 0.001
CVD	37/479	50/680	1.56 (0.95-2.55)	> 0.05
Cancer	28/479	50/680	0.79 (0.35-1.79)	> 0.05

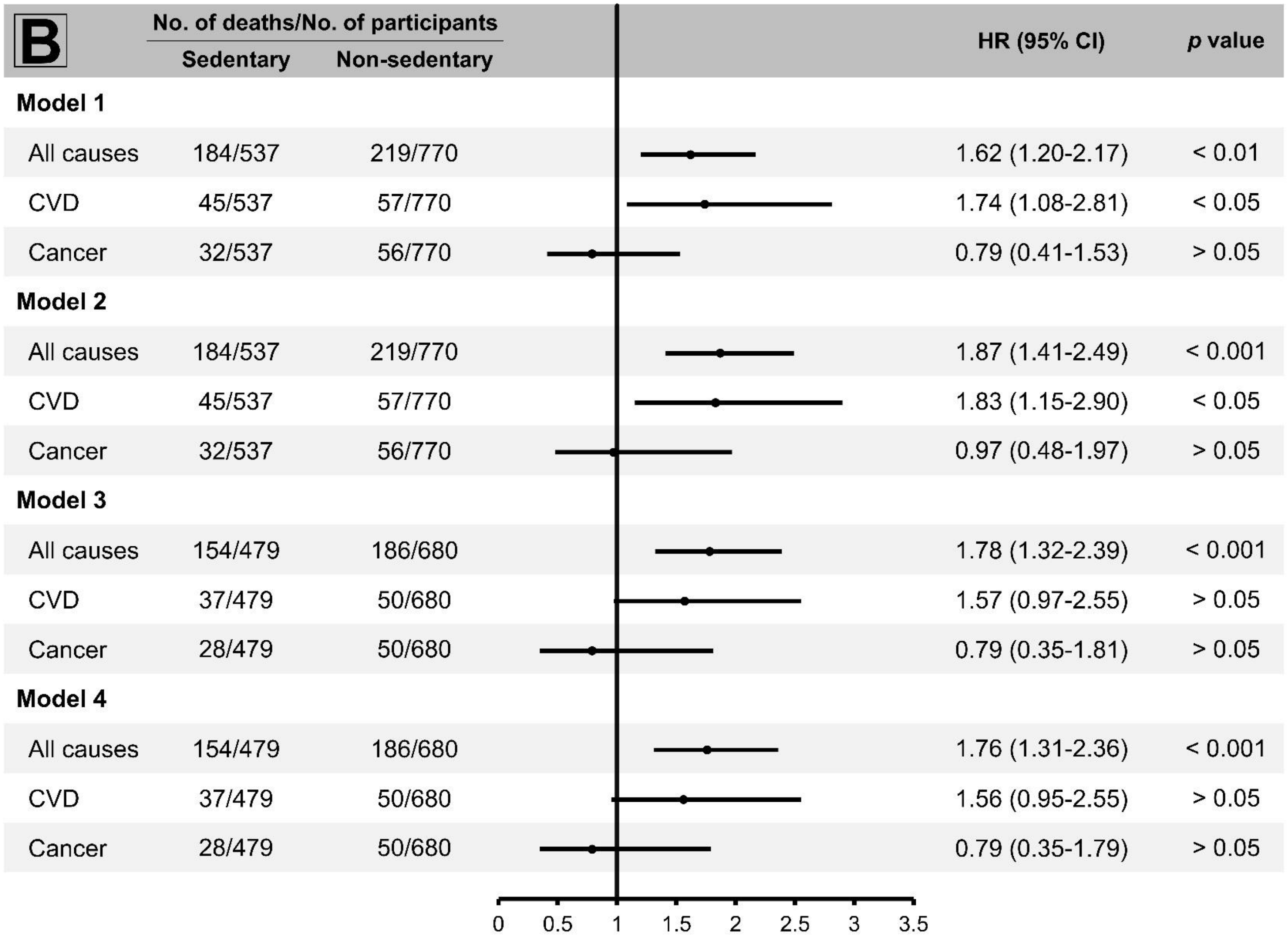

Supplemental Figure S1C

C

	No. of deaths/No. of participants		HR (95% CI)	*p* value
	Sedentary	Non-sedentary		
**Model 1**				
All causes	165/452	176/610	1.62 (1.13-2.34)	< 0.01
CVD	39/452	41/610	1.72 (0.95-3.13)	> 0.05
Cancer	32/452	44/610	0.96 (0.47-1.97)	> 0.05
**Model 2**				
All causes	165/452	176/610	1.93 (1.34-2.78)	< 0.001
CVD	39/452	41/610	1.79 (0.98-3.26)	> 0.05
Cancer	32/452	44/610	1.24 (0.56-2.75)	> 0.05
**Model 3**				
All causes	130/391	154/540	1.67 (1.16-2.39)	< 0.01
CVD	28/391	37/540	1.22 (0.64-2.32)	> 0.05
Cancer	28/391	41/540	1.07 (0.42-2.72)	> 0.05
**Model 4**				
All causes	130/391	154/540	1.52 (1.07-2.14)	< 0.05
CVD	28/391	37/540	1.14 (0.58-2.26)	> 0.05
Cancer	28/391	41/540	0.98 (0.41-2.34)	> 0.05

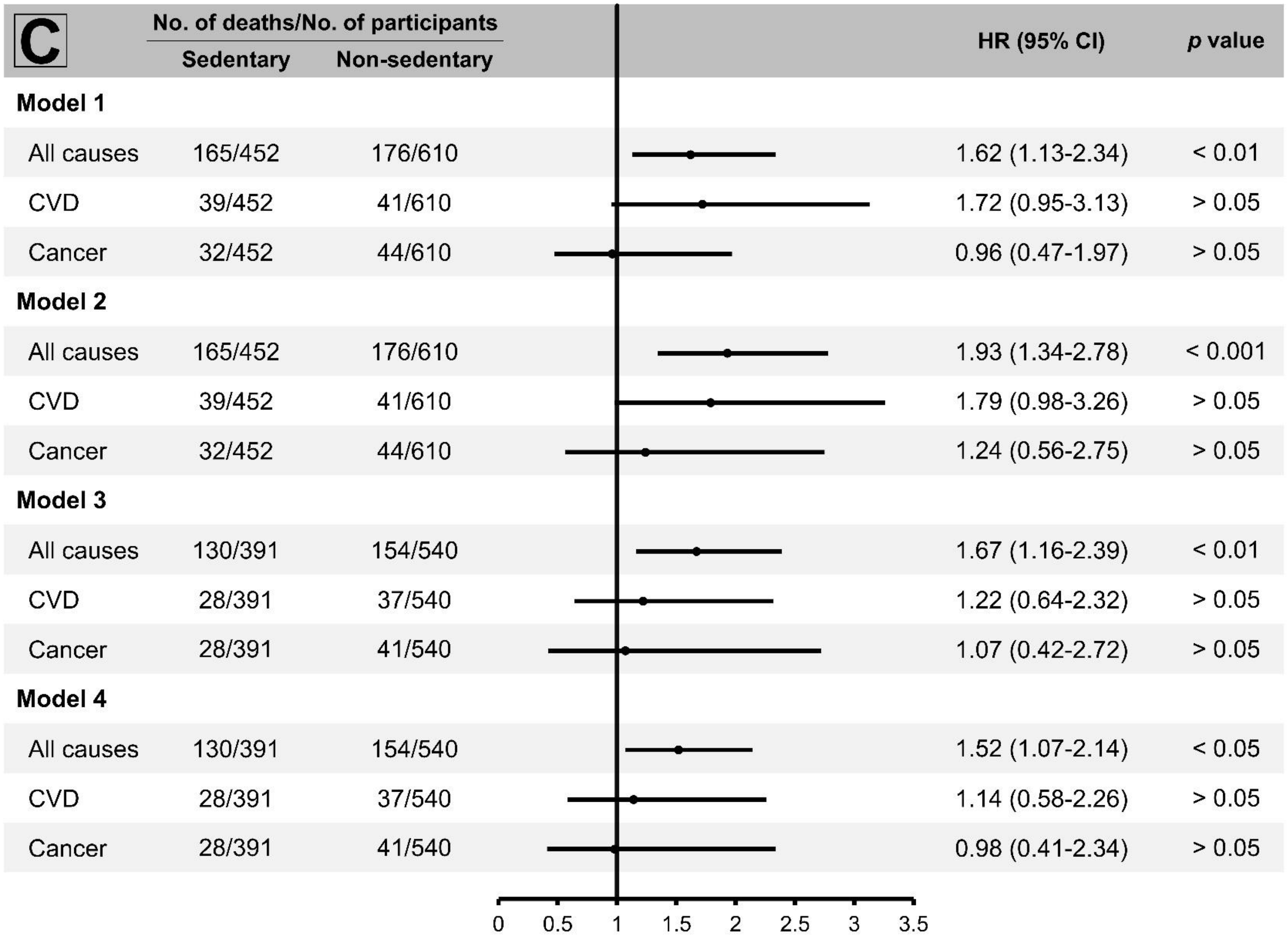

Supplemental Figure S1D

D	No. of deaths/No. of participants		HR (95% CI)	*p* value
	Sedentary	Non-sedentary		
**Model 1**				
All causes	58/272	119/470	0.90 (0.60-1.36)	> 0.05
CVD	12/272	24/470	0.70 (0.27-1.81)	> 0.05
Cancer	12/272	36/470	0.46 (0.20-1.06)	> 0.05
**Model 2**				
All causes	58/272	119/470	1.18 (0.78-1.79)	> 0.05
CVD	12/272	24/470	0.72 (0.28-1.85)	> 0.05
Cancer	12/272	36/470	0.61 (0.24-1.58)	> 0.05
**Model 3**				
All causes	56/253	110/428	1.30 (0.83-2.01)	> 0.05
CVD	11/253	22/428	0.82 (0.29-2.31)	> 0.05
Cancer	12/253	35/428	0.53 (0.19-1.52)	> 0.05
**Model 4**				
All causes	56/253	110/428	1.22 (0.77-1.94)	> 0.05
CVD	11/253	22/428	0.75 (0.31-1.78)	> 0.05
Cancer	12/253	35/428	0.49 (0.17-1.41)	> 0.05

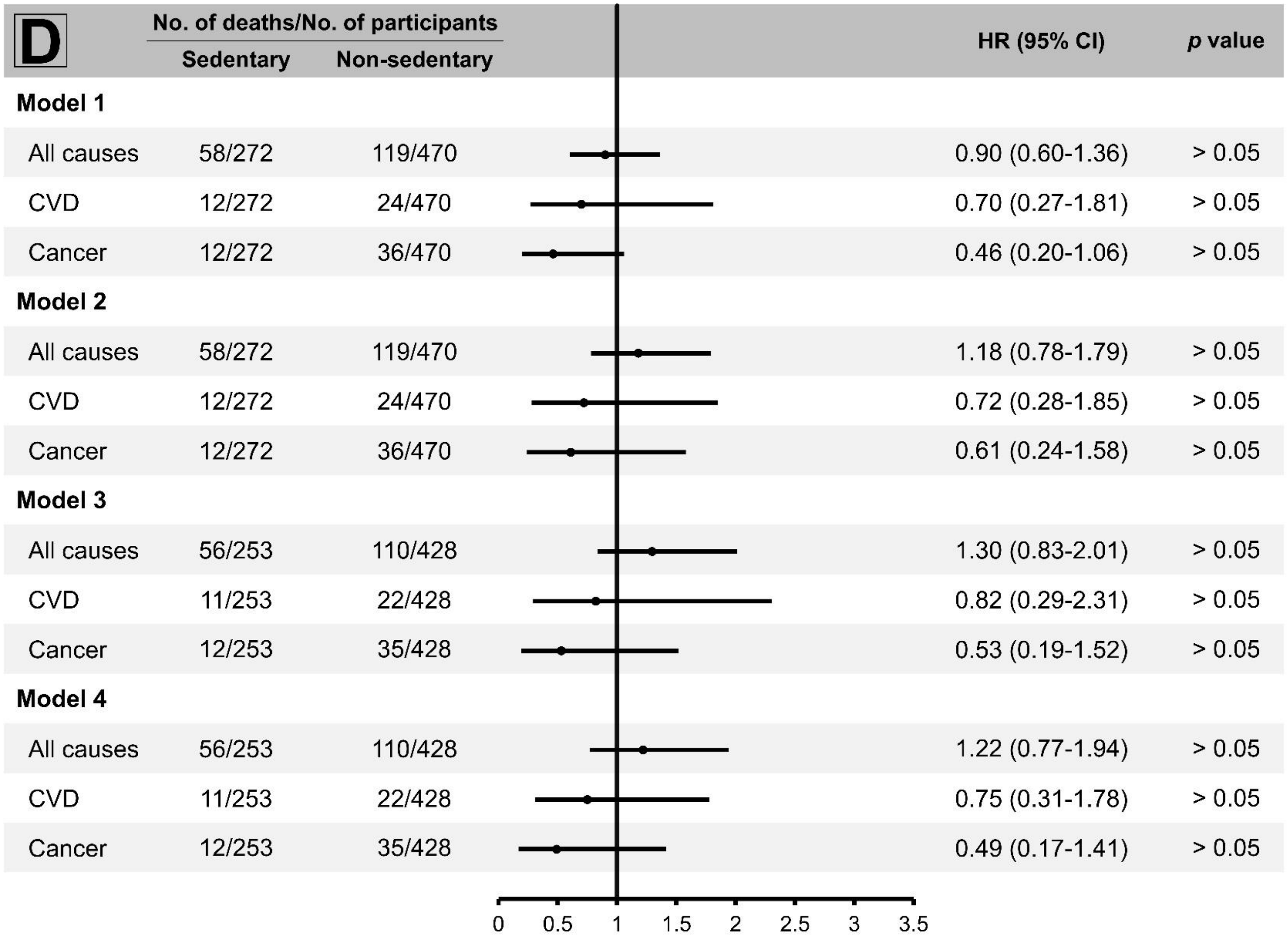

Supplement: SUPPLEMENTARY MATERIAL [file kidney360-5-033-s001.pdf]
